# Supplementary material for: Statins and renal disease progression, ophthalmic manifestations, and neurological manifestations in veterans with diabetes: A retrospective cohort study
Source: PLoS One. 2022 Jul 21;17(7):e0269982. doi: 10.1371/journal.pone.0269982 (PMC9302779; doi:10.1371/journal.pone.0269982)
Supplement: S1 File — (DOCX) [file pone.0269982.s001.docx]

**Supplementary online material**

**Statins and risk of renal, ophthalmic, and neurological complications in Veterans with diabetes: A retrospective cohort study**

**Ishak A. Mansi, MD; Matheu Chansard; Ildiko Lingvay, MD, MPH, MSCS; Song Zhang, PhD; Ethan A. Halm, MD, MPH, MBA ; Carlos A Alvarez, PharmD, MSc, MSCS**

- S-Methods: ……………………………………………………………………….. Page 2
  - CDW
  - Protocol for laboratory tests and vital signs handling
  - Propensity score matching details
  - S1 Fig. Propensity scores before matching
  - S2 Fig. Propensity scores after matching
- S1 Table. Definition of administrative codes used in the study outcomes ……… Page 4
- S2 Table. Administrative codes used in definitions of baseline characteristics … Page 6
- S3 Table. Secondary analysis definitions ……………………………………… Page 10
- S3 Fig. Study design and cohort assembly………………………………….... Page 11
- S4 Table. Baseline characteristics of statin users and active comparators in the

overall cohort before propensity score matching ………………………………. Page 12

- S5 Table. Comparisons of changes in vital signs and laboratory values

in propensity score matched cohort of statin users and nonusers ……………… Page 18

- S6 Table. Summary of the major placebo controlled randomized cardiovascular

outcome trials evaluating statins in a primary prevention in patients with diabetes Page 19

**S-Methods**

- **CDW**

The national VA Corporate Data Warehouse (CDW) encompasses inpatient and outpatient diagnosis/procedure codes, pharmacy, vital sign, and laboratory data. CDW catalogues its data according to published protocols (reference 25 in the manuscript).

- **Protocol for laboratory tests and vital signs handling**

Laboratory values were captured using 2 different techniques: 1) searching the name of the test in the laboratory database and 2) using LOINC codes that were known to be at utilization in the VA at its time, as the followings.

Hemoglobin A1C (Glycohemoglobin): 17855-8, 17856-6, 4548-4, 4549-2

LDL-Cholesterol (both calculated and direct): 13457-7, 18262-6, 2089-1, 49132-4

HDL-Cholesterol: 2085-9

Total Cholesterol: 2093-3

Serum Creatinine: 2160-0

Duplicate values were identified and removed. Extreme values were discarded. Overall, only 0.21% of the laboratory investigation values were identified as extreme and discarded.

eGFR was calculated using MDRD formula (reference 30 and 31 in manuscript):

GFR, in mL/min per 1.73 m2 = 175 x SCr (exp[-1.154]) x Age (exp[-0.203]) x (0.742 if female) x (1.21 if black)

Stage 5 Chronic Kidney Disease was defined as an incident decrease in mean estimated glomerular filtration rate (eGFR) during the last year of follow up to <15 mL/min/1.73m^2^ (stage 5)-(reference 30 and 31 in manuscript).

If race was missing, we considered it white for purpose of calculating eGFR for both baseline and follow-up.

Similarly, extreme values for weight, systolic blood pressure, and diastolic blood pressure were discarded. Overall, 0.0007% of the values were identified as extreme and discarded.

- **Propensity score matching details**

We created a propensity score to match statin-users and active comparators (nonusers) at a ratio of 1:1 using 99 variables chosen *a priori*, which comprised: Age, gender (self-reported), race and ethnicity (self-reported), demographics, personal history, vital signs, comorbidities, comorbidity and cardiovascular scores,[_ENREF_42](#_ENREF_42) occurrence of outcomes of interest at baseline, healthcare utilization, laboratory values, and glucose-lowering medication classes and non-diabetes medication classes (Table 1 in the manuscript). All baseline characteristics were selected *a priori*. We used the routine by Leuven and Sianesi to perform nearest number matching using the logit model with no replacement (references 55 and 56 in the manuscript). We explored a caliper width of 0.01, which approximately represented 0.2 times the standard deviation of the logit of the propensity scores, as suggested in prior publications (Austin PC, *Pharm Stat.* Mar-Apr 2011;10(2):150-161). We subsequently decreased caliper by a decrement of 10% and checked standardized differences on all covariates. Using smaller caliper, we improved balance with < 10% decrease in sample size. A caliper of 0.0008 was found to offer best balance in differences, without residual statistically significant differences between treatment groups on most covariates, specifically, most important variables such as proportion of diabetes and its complications at baseline, duration of follow up, and other measures of healthcare utilization. After propensity score creation, pseudo R2 decreased to 0.001, indicating that successful balance has been achieved (Staffa et al. *Anesth Analg.* Oct 2018;127(4):1066-1073). Graph 1 and graph 2 depict kernel graphs of propensity score before and after matching, respectively.


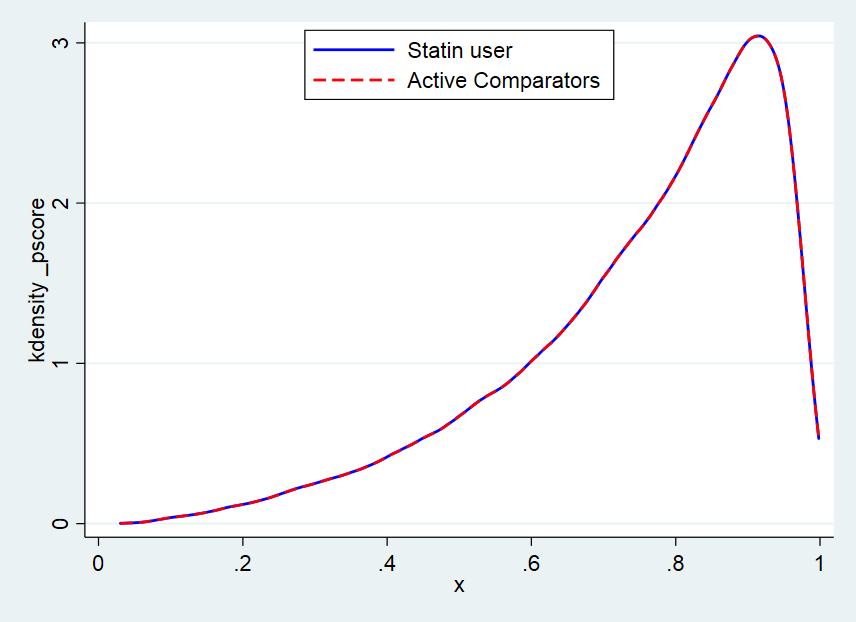


S2 Fig. Propensity scores after matching


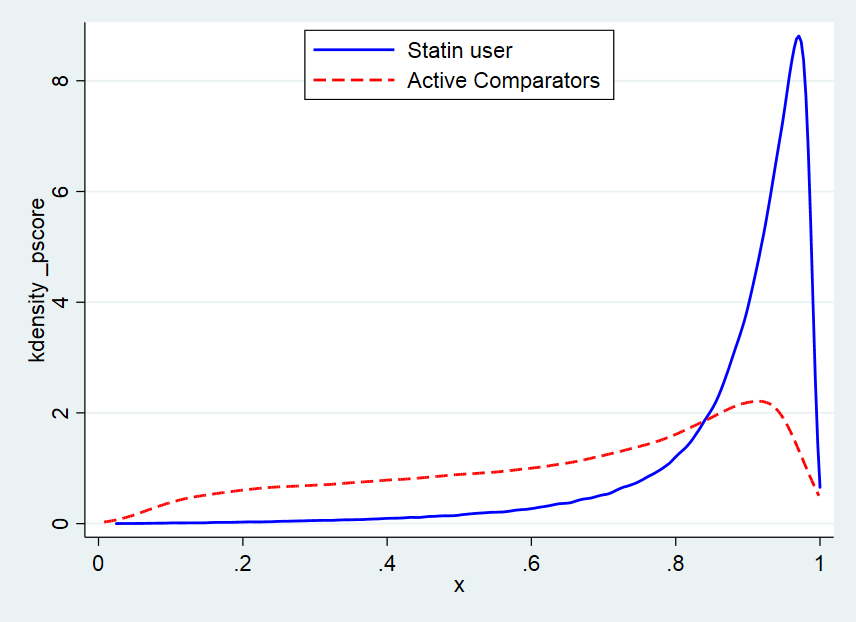


S1 Fig. Propensity scores before matching

| **S1 Table. Definition of administrative codes used in the study outcomes** | |
| --- | --- |
| Diabetes with renal manifestations: AHRQ-CCS category 3.3.2 | 24940 24941 25040 25041 25042 25043 |
| Diabetes with ophthalmic manifestations: AHRQ-CCS category 3.3.3 | 24950 24951 25050 25051 25052 25053 |
| Diabetes with neurological manifestations: AHRQ-CCS category 3.3.4 | 24960 24961 25060 25061 25062 25063 |
| Nephritis; nephrosis; renal sclerosis: AHRQ-CCS category 156 & Chronic kidney disease: AHRQ-CCS category 158 | 5800 5804 58081 58089 5809 5810 5811 5812 5813 58181 58189 5819 5820 5821 5822 5824 58281 58289 5829 5830 5831 5832 5834 5836 5837 58381 58389 5839 587  &  585 5851 5852 5853 5854 5855 5856 5859 7925 V420 V451 V4511 V4512 V560 V561 V562 V5631 V5632 V568 |
| Renal replacement therapy: these codes were used in the literature and found to have high sensitivity and specificity (references 32 and 33 in the manuscript) | ICD-9-CM diagnosis codes: 585.6 (End stage renal disease) 585.xx (dialysis) V56.0 (Extracorporeal dialysis) V56.8 (Other dialysis) 5561 5569 (AHRQ-CCS multilevel category 10.4: Kidney transplant)  ICD9 procedure codes: 39.95 (Hemodialysis) 54.98 (Peritoneal dialysis) 55.69 (Other kidney transplantation)  CPT codes: 90953 90954 90953 90954 90955 90956 90957 90958 90959 90960 90961 90962  90963 90964 90965 90966 90967 90968 90969 90970 90989 90993 90999 50365 50360 |
| Chronic obstructive pulmonary disease (COPD), as defined using ICD-9-CM codes by Deyo et al | 490-496 500-505 5064 |
| Suicide and intentional self-inflicted injury, as defined using ICD-9-CM codes (AHRQ-CCS category 662). | E9500 E9501 E9502 E9503 E9504 E9505 E9506 E9507 E9508 E9509 E9510 E9511 E9518 E9520 E9521 E9528 E9529 E9530 E9531 E9538 E9539 E954 E9550 E9551 E9552 E9553 E9554 E9555 E9556 E9557 E9559 E956 E9570 E9571 E9572 E9579 E9580 E9581 E9582 E9583 E9584 E9585 E9586 E9587 E9588 E9589 E959 V6284 |
| Any retinopathy and its complications: These codes were used in the literature and were found to have high positive and negative predictive value for diagnosis of diabetic retinopathy,[_ENREF_39](#_ENREF_39) and sensitivity of 88% and a specificity of 96% for identifying diabetic macular edema (manuscripts 29,33, and 60 in the manuscript). | 362.0x (Diabetic retinopathy) 362.01, 362.03, 362.04, 362.05, 362.06 (Non-proliferative DR)  362.02 (Proliferative DR) 379.23 (vitreous hemorrhage) 362.81 (Retinal hemorrhage) 362.82 (Retina exudates/deposits) 362.83 (Retinal edema) 362.84 (Retinal ischemia) 362.85 (Retinal nerve fiber defects) 362.89 (Retinal disorders NEC) 362.81 (Vitreous hemorrhage) 361.0x 361.8x 361.9 (retinal detachment not specific DM) 362.07 (Diabetic macular edema) 362.53 (Cystoid macular degeneration) |

| **S2 Table. Administrative codes used in definitions of baseline characteristics** | |
| --- | --- |
| **Baseline characteristic** | **Definition** |
| Family history of cardiovascular diseases | ICD-9-CM codes: V171, V1749, V174, V1741, and V173. |
| Smoking | ICD-9-CM codes: 3051 and V1582 |
| Alcohol-related disorders | AHRQ-CCS category 660 |
| Substance-related disorders | AHRQ-CCS category 661 |
| Obesity: Diagnosis of obesity by ICD-9 codes have a very high specificity (≈98%) but low sensitivity (≈30%). (Quan et al. Health Serv Res. 2008;43(4):1424-1441 & Goff et al. Paediatr Perinat Epidemiol. Sep 2012;26(5):421-429) | ICD-9-CM diagnosis codes from category 56 of AHRQ-CCS (other nutritional; endocrine; and metabolic disorders) related to overweight, obesity and hyperalimentation (codes: 2780, 27800, 27801, 27802, 27803, 2781, 2788, and 7831). |
| Received immunization and infectious disease screening | AHRQ-CCS category 10  CPT codes: 90281 90287 90291 90296 90371 90375 90376 90378 0379 90389 90393 90396 90470 90476 90585 90620 90621 90630 90477 90581 90632 90633 90634 90636 90644 90645 90646 90647 90648 90649 90650 90651 90654 90655 90656 90657 90658 90659 90660 90661 90662 90663 90664 90666 90668 90669 90670 90672 90673 90675 90676 90680 90681 90685 90686 90687 90688 90690 90691 90692 90693 90696 90698 90700 90700 90701 90702 90703 90704 90705 90706 90707 90708 90710 90712 90713 90714 90714 90715 90716 90717 90718 90720 90721 90723 90724 90725 90726 90727 90728 90730 90731 90732 0733 90734 90734 90735 90736 90737 90738 90740 90741 90743 90744 90745 90746 90747 90748" |
| Received rehabilitation care; fitting of prostheses; and adjustment of devices | AHRQ-CCS category 254 |
| Diabetes mellitus without complications | AHRQ-CCS category 49 |
| Diabetes mellitus with complications | AHRQ-CCS category 50 |
| Diabetes with ketoacidosis or uncontrolled diabetes | AHRQ-CCS category 3.3.1 and Selected codes from AHRQ-CCS category 3.3.7 (24920 24921 24930 24931 25020 25021 25022 25023 25030 25032 25033) |
| Diabetes with circulatory manifestations | AHRQ-CCS category 3.3.5 |
| Diabetes with unspecified complications | AHRQ-CCS category 3.3.6 |
| Peripheral Ulcer | 443.81 707.10-707.19 707.8 707.9 731.8 |
| Diabetic foot | 250.70 250.71 250.72 250.73 250.7x 785.4 |
| Any retinopathy & its complications | Refer to outcomes definitions |
| Any neuropathy: These codes were used at the literature in various combinations (Kang EY et al. *JAMA Ophthalmol.* 2019;137(4):363-371; Jung HK et al. Gastroenterology;136(4):1225-1233; and Hyett B et a. Gastroenterology 2009;137(2):445-452) | 536.3 (gastroparesis) 354.1-355.9 (mononeuropathy of any reason excluding carpal tunnel syndrome) 337.1 (Peripheral autonomic neuropathy in disorders classified elsewhere) 357.2 (Polyneuropathy in diabetes) 536.3 (Stomach function dis NEC) 536.8 (Stomach function dis NEC) 250.6X (diabetes with neurologic manifestations) |
| Valvular heart disease | AHRQ-CCS category 96 |
| Pericarditis, endocarditis, myocarditis, or cardiomyopathy | AHRQ-CCS category 97 |
| Hypertension | AHRQ-CCS category 98 |
| Hypertension with complications and secondary hypertension | AHRQ-CCS category 99 |
| Acute myocardial infarction | AHRQ-CCS category 100 |
| Coronary atherosclerosis and other heart disease | AHRQ-CCS category 101 |
| Nonspecific chest pain | AHRQ-CCS category 102 |
| Heart disease not otherwise specified | AHRQ-CCS category 104 |
| Pulmonary heart disease | AHRQ-CCS category 103 |
| Conduction disorders | AHRQ-CCS category 105 |
| Cardiac dysrhythmias | AHRQ-CCS category 106 |
| Cardiac arrest and ventricular fibrillation | AHRQ-CCS category 107 |
| Congestive heart failure; non-hypertensive | AHRQ-CCS category 108 |
| Acute cerebrovascular disease | AHRQ-CCS category 109 |
| Occlusion or stenosis of precerebral arteries | AHRQ-CCS category 110 |
| Other and ill-defined cerebrovascular disease | AHRQ-CCS category 111 |
| Transient cerebral ischemia | AHRQ-CCS category 112 |
| Peripheral and visceral atherosclerosis | AHRQ-CCS category 114 |
| Aortic; peripheral; and visceral artery aneurysms | AHRQ-CCS category 115 |
| Aortic and peripheral arterial embolism or thrombosis | AHRQ-CCS category 116 |
| Chronic obstructive pulmonary disease and bronchiectasis | AHRQ-CCS category 127 |
| Asthma | AHRQ-CCS category 128 |
| Respiratory failure; insufficiency; arrest in adult | AHRQ-CCS category 131 |
| Acute and unspecified renal failure | AHRQ-CCS category 157 |
| Rheumatoid arthritis and related disease; Systemic lupus erythematosus and connective tissue disorders | AHRQ-CCS category 202 & 210 |
| Pathological fracture | AHRQ-CCS category 207 |
| schizophrenia and other psychotic disorders | AHRQ-CCS category 659 |
| suicide and intentional self-inflicted injury | AHRQ-CCS category 662 |
| Severe liver disease* | ICD-9 codes: 572.2-572.8 |
| Malignancy* | ICD-9 codes: 140-172.9; 174-195.8 |
| Metastatic neoplasm* | ICD-9 codes: 196-199.1 |
| Acquired Immunodeficiency Syndrome* | ICD-9 codes: 042-044.9 |
| Charlson Comorbidity Index | Using Deyo’s method[^1^](#_ENREF_1) |
| cardiovascular risk | using D’ Agostino method to calculate the Framingham score[_ENREF_12](#_ENREF_12) (D'Agostino RB et al. Circulation 2008;117(6):743-753) |
| AHRQ-CCS = the Agency for Health Research and Quality Clinical Classifications Software  *Malignancy, metastatic neoplasm, and Acquired Immunodeficiency Syndrome were defined using Deyo et al method in calculating the Charlson comorbidity index. | |

| **S3 Table. Secondary analysis definitions** | |
| --- | --- |
| The Overall cohort | Included all eligible patients before propensity score matching |
| Healthy cohort | Included only patients with a Charlson comorbidity index of zero at baseline |
| Intensive cholesterol lowering statin users in comparison to nonusers in the overall cohort | Intensive cholesterol lowering statin was defined as a decrease of ≥50% in mean LDL-cholesterol during follow-up in comparison to the mean during baseline. This definition was guided by the American College of Cardiology /American Heart Association (ACC/AHA) definition of high-intensity statin therapy [(reference](#_ENREF_17) 1 in the manuscript) |
| Medium-intensity cholesterol lowering statin users in comparison to nonusers in the overall cohort | Medium-intensity statin was defined as a decrease of <50% and ≥30% in mean LDL-cholesterol during follow-up in comparison to baseline. |
| Low-intensity cholesterol lowering statin users in comparison to nonusers in the overall cohort | Low-intensity statin was defined as a decrease of <30% in mean LDL-cholesterol during follow-up in comparison to baseline |
| Time-to-event analysis in the propensity score matched cohort: | We estimated the hazard ratio (HR) in statin users in comparison to nonusers using survival regression analysis of the following outcomes: a) Incident CKD; b) Incident diabetes with ophthalmic manifestations; and c) Incident diabetes with neurological manifestations. We performed a separate regression analysis for each of these outcomes. |
| Time-to-event analysis in the propensity score matched cohort with death as a competing risk | We estimated the subhazard ratio (SHR) in statin users in comparison to nonusers using survival regression analysis using similar outcomes to previous analysis |

**S3 Fig. Study design and cohort assembly**

53,373,845 statin prescriptions and 7,575,663‬ H2 or PPI prescriptions between study times for the initial cohort

Excluded 1098 patients with missing Date of Birth


**Active comparators**

N = 110,195

**Active comparators**

N = 81,146

**Statin users**

N = 81,146

**Statin users**

N = 595,579

Propensity score matching at a ratio of 1:1

Patients with complete data 705,774

**Incident Statin users**

N=910,337

Excluded:

- 3236 age <30 year-old
- 5,847 follow-up <60 days
- 305,536 with incomplete lab or vital signs data

**Active comparators**

N=851,886 patients

Excluded:

- 436,359 concomitant use of statins (counted as statin users)
- 156,021 prevalent H2-PPI users

**Statin users**

N=1,194,996 patients

Excluded:

- 285,757 prevalent users

**Incident active comparators**

N = 259,506

Excluded:

- 3377 age <30 year-old
- 30,238 follow-up <60 days
- 115,634 with incomplete lab or vital signs data

| **S4 Table. Baseline characteristics of statin users and active comparators in the overall cohort before propensity score matching** | | | |
| --- | --- | --- | --- |
|  | **Statin users**  **(n =** **595,579 )** | **Active comparators**  **(n =** **110,195)** | **p-value** |
| **Baseline characteristics included in propensity score** | | | |
| Age at index date (years): mean (SD) | 60.7 (10.5) | 59.3 (11.6) | <0.001 |
| Male Gender | 573,455 (96.3) | 104,235 (94.6) | <0.001 |
| Race |  |  |  |
| Caucasian | 426,253 (71.6) | 74,946 (68.0) | <0.001 |
| African American | 108,178 (18.2) | 24,378 (22.1) | <0.001 |
| American Indians/Alaskan, pacific/Hawaiian | 12,170 (2.0) | 2,389 (2.2) | <0.001 |
| Asian | 4,543 (0.8) | 633 (0.6) | <0.001 |
| Unknown/missing | 44,435 (7.5) | 7,849 (7.1) | <0.001 |
| Ethnicity |  |  |  |
| Hispanic/Latino | 37,152 (6.2) | 7,648 (6.9) | <0.001 |
| Non-Hispanic/Latino | 529,171 (88.9) | 97,360 (88.4) | <0.001 |
| Unknown/missing | 29,256 (4.9) | 5,187 (4.7) | 0.004 |
| **Social and family history during baseline period** |  |  |  |
| Family history of cardiovascular diseases^1^ | 9,743 (1.6) | 1,262 (1.2) | <0.001 |
| Smoking^2^ | 110,710 (18.6) | 22,723 (20.6) | <0.001 |
| Alcohol-related disorders^3^ | 35,133 (5.9) | 11,800 (10.7) | <0.001 |
| Substance-related disorders^3^ | 25,123 (4.2) | 8,682 (7.9) | <0.001 |
| **Vital data during baseline period** |  |  |  |
| Mean systolic blood pressure (mmHg): mean (SD) | 137 (15) | 135 (14) | <0.001 |
| Mean diastolic blood pressure (mmHg): mean (SD) | 79 (10) | 78 (10) | <0.001 |
| Body mass index |  |  |  |
| < 25 kg/m^2^ | 50,118 (8.4) | 14,233 (12.9) | <0.001 |
| 25 to <30 kg/m^2^ | 161,330 (27.1) | 30,631 (27.8) | <0.001 |
| 30 to <35 kg/m^2^ | 166,751 (28.0) | 28,158 (25.6) | <0.001 |
| 35 to <40 kg/m^2^ | 86,780 (14.6) | 14,472 (13.1) | <0.001 |
| 40 to <45 kg/m^2^ | 33,530 (5.6) | 5,547 (5.0) | <0.001 |
| ≥ 45 kg/m^2^ | 17,983 (3.0) | 3,065 (2.8) | <0.001 |
| Missing | 79,087 (13.3) | 14,089 (12.8) | <0.001 |
| **Healthcare utilization during baseline period** | | | |
| Number of inpatient admissions: |  |  |  |
| mean (SD) | 0.83 (2.95) | 1.54 (4.03) | <0.001 |
| median (interquartile) | 0 (0, 0) | 0 (0, 0) | <0.001 |
| Number of outpatient encounters |  |  |  |
| mean (SD) | 9.6 (15.7) | 12.6 (20.6) | <0.001 |
| median (interquartile) | 5 (2, 11) | 7 (3, 15) | <0.001 |
| Received immunization and infectious disease screening | 237,284 (39.8) | 42,705 (38.8) |  |
| Received rehabilitation care; fitting of prostheses; and adjustment of devices | 40,612 (6.8) | 11,345 (10.3) | <0.001 |
| **Diabetes and its complications during baseline period:^3^** | | | |
| Diabetes mellitus | 371,324 (62.4) | 51,136 (46.4) | <0.001 |
| Diabetes with complications | 82,454 (13.8) | 11,964 (10.9) | <0.001 |
| Diabetes with ketoacidosis or uncontrolled diabetes | 33,222 (5.6) | 4,534 (4.1) | <0.001 |
| Diabetes with renal manifestations | 7,032 (1.2) | 934 (0.9) | <0.001 |
| Diabetes with ophthalmic manifestations | 12,933 (2.2) | 1,943 (1.8) | <0.001 |
| Diabetes with neurological manifestations | 28,536 (4.8) | 4,524 (4.1) | <0.001 |
| Diabetes with circulatory manifestations | 2,575 (0.4) | 405 (0.4) | 0.002 |
| Diabetes with unspecified manifestations | 6,522 (1.1) | 862 (0.8) | <0.001 |
| Diabetic foot^4^ | 3,112 (0.5) | 546 (0.5) | 0.25 |
| Peripheral ulcer^4^ | 7,688 (1.3) | 1,922 (1.7) | <0.001 |
| Below knee amputations^4^ | 34 (0.01) | 6 (0.0) | 0.92 |
| Above knee amputations^4^ | 5 90.0) | 0 (0.0) | 0.34 |
| Any retinopathy & its complications^4^ | 19,392 (3.3) | 3,196 (2.9) | <0.001 |
| **Other comorbidities during baseline period:^3^** | | | |
| Obesity as defined by ICD-9 codes^5^ | 148,522 (24.9) | 24,267 (22.0) | <0.001 |
| Valvular heart disease | 15,811 (2.7) | 2,958 (2.7) | 0.58 |
| Peri-; endo-; and myocarditis; cardiomyopathy | 8,129 (1.4) | 1,321 (1.2) |  |
| Hypertension | 416,803 (70.0) | 68,260 (61.9) | <0.001 |
| Hypertension with complication or secondary hypertension | 12,371 (2.1) | 2,309 (2.1) | 0.70 |
| Acute myocardial infarction | 6,298 (1.1) | 261 (0.2) | <0.001 |
| Coronary atherosclerosis and other heart disease | 114,541 (19.2) | 11,505 (10.4) | <0.001 |
| Nonspecific chest pain | 39,836 (6.7) | 7,977 (7.2) | <0.001 |
| Pulmonary heart disease | 4,437 (0.7) | 943 (0.9) | <0.001 |
| Other and ill-defined heart disease | 10,450 (1.8) | 1,757 (1.6) | <0.001 |
| Conduction disorders | 12,021 (2.0) | 2,035 (1.9) | <0.001 |
| Cardiac dysrhythmias | 46,382 (7.8) | 8,836 (8.0) | 0.01 |
| Cardiac arrest and ventricular fibrillation | 297 (0.05) | 47 (0.04) | 0.32 |
| Congestive heart failure | 25,932 (4.4) | 4,136 (3.8) | <0.001 |
| Acute cerebrovascular disease | 17,628 (3.0) | 2,095 (1.9) | <0.001 |
| Occlusion or stenosis of precerebral arteries; ill-defined cerebrovascular disease; Transient cerebral ischemia | 12,402 (2.1) | 1,450 (1.3) | <0.001 |
| Peripheral and visceral atherosclerosis | 23,125 (3.9) | 3,164 (2.9) | <0.001 |
| Aortic; peripheral; and visceral artery aneurysms | 5,589 (0.9) | 883 (0.8) | <0.001 |
| Aortic and peripheral arterial embolism or thrombosis | 936 (0.2) | 168 (0.2) | 0.72 |
| Chronic obstructive pulmonary disease and bronchiectasis | 60,682 (10.2) | 13,590 (12.3) | <0.001 |
| Asthma | 21,187 (3.6) | 5,011 (4.6) | <0.001 |
| Respiratory failure; insufficiency; arrest | 2,582 (0.4) | 793 (0.7) | <0.001 |
| Nephritis; nephrosis; renal sclerosis; Chronic kidney disease | 21,702 (3.6) | 4,084 (3.7) | 0.31 |
| Acute and unspecified renal failure | 8,714 (1.5) | 2,514 (2.3) | <0.001 |
| Renal replacement therapy | 5,652 (1.0) | 1,511 (1.4) | <0.001 |
| Rheumatoid arthritis; Systemic lupus erythematosus and connective tissue disorders | 6,226 (1.1) | 1,840 (1.7) | <0.001 |
| Pathological fracture | 290 (0.05) | 129 (0.1) | <0.001 |
| Schizophrenia and other psychotic disorders | 15,130 (2.5) | 4,178 (3.8) | <0.001 |
| Suicide and intentional self-inflicted injury | 3,617 (0.6) | 1,240 (1.1) | <0.001 |
| Severe liver disease^6^ | 945 (0.2) | 1,394 (1.3) | <0.001 |
| Malignancy^6^ | 39,941 (6.7) | 9,714 (8.8) | <0.001 |
| Metastatic neoplasm^6^ | 1,226 (0.2) | 709 (0.6) | <0.001 |
| Acquired Immunodeficiency Syndrome^6^ | 1,872 (0.3) | 766 (0.7) | <0.001 |
| Any neuropathy^4^ | 130,579 (21.9) | 15,549 (14.1) | <0.001 |
| **Comorbidity Score** |  |  |  |
| Charlson Comorbidity Total Score^7^: |  |  |  |
| mean (SD) | 1.29 (1.25) | 1.27 (1.50) | <0.001 |
| median (interquartile) | 1 (1, 2) | 1 (0, 2) | <0.001 |
| Cardiovascular risk^8^ |  |  |  |
| < 5% | 84,226 (14.1) | 30,138 (27.4) | <0.001 |
| 5 to <10% | 103,753 (17.4) | 21,310 (19.3) | <0.001 |
| 10 to <15% | 146,763 (24.6) | 24,217 (22.0) | <0.001 |
| 15 to <20% | 141,990 (23.8) | 19,215 (17.4) | <0.001 |
| 20 to <25% | 76,949 (12.9) | 8,712 (7.9) | <0.001 |
| 25 to <30% | 19,921 (3.3) | 1,884 (1.7) | <0.001 |
| ≥30% | 1,895 (0.3) | 151 (0.1) | <0.001 |
| Missing | 20,082 (3.4) | 4,568 (4.2) | <0.001 |
| **Laboratory investigations** |  |  |  |
| Mean glucose in blood in mg/dL: mean (SD) | 142 (57) | 130 (48) | <0.001 |
| Mean serum creatinine in mg/dL: mean (SD) | 1.10 (0.4) | 1.10 (0.62) | 0.02 |
| At least one blood glucose of 200mg/dL or more | 133,872 (22.5) | 20,994 (19.1) | <0.001 |
| More than 5 measurements with blood glucose of 200mg/dL or more | 22,384 (3.8) | 5,558 (5.0) | <0.001 |
| Mean eGFR |  |  |  |
| >90 mL/min per 1.73 m^2^ | 139,503 (23.4) | 30,237 (27.4) | <0.001 |
| 60 to 89 mL/min per 1.73 m^2^ | 343,528 (57.7) | 61,098 (55.5) | <0.001 |
| 45 to 59 mL/min per 1.73 m^2^ | 80,060 (13.4) | 12,855 (11.7) | <0.001 |
| 30 to 44 mL/min per 1.73 m^2^ | 24,484 (4.1) | 4,104 (3.7) | <0.001 |
| 15 to 29 mL/min per 1.73 m^2^ | 5,939 (1.0) | 1,205 (1.1) | 0.003 |
| <15 mL/min per 1.73 m^2^ | 2,065 (0.35) | 696 (0.6) | <0.001 |
| Mean eGFR: mean (SD) | 76.93 (21.18) | 79.07 (23.01) | <0.001 |
| **Glucose lowering medications classes^6^** |  |  |  |
| Metformin | 145,324 (24.4) | 18,169 (16.5) | <0.001 |
| Sulphonylurea | 98,344 (16.5) | 13,440 (12.2) | <0.001 |
| GLP1 | 149 (0.03) | 19 (0.02) | 0.12 |
| DDP4 | 649 (0.1) | 98 (0.1) | 0.06 |
| Thiazolidinediones | 13,460 (2.3) | 1,483 (1.4) |  |
| α-glucosidase inhibitors | 28 (0.0) | 1 (0.0) | 0.07 |
| Amylin analog | 20 (0.0) | 3 (0.0) | 0.73 |
| SGLT2 | 2 (0.0) | 0 (0.0) | 0.54 |
| Insulins | 7,834 (7.1) | 56,007 (9.4) | <0.001 |
| Total number of anti-diabetes medication groups: |  |  |  |
| mean (SD) | 0.53 (0.79) | 0.37 (0.71) | <0.001 |
| Median (interquartile) | 0 (0, 1) | 0 (0, 1) | <0.001 |
| **Other medications groups** |  |  |  |
| ACEI | 214,428 (36.0) | 33,482 (30.4) | <0.001 |
| ARB | 31,076 (5.2) | 5,200 (4.7) | <0.001 |
| Beta-blockers | 133,168 (22.4) | 21,385 (19.4) | <0.001 |
| Non-loop diuretic | 148,691 (25.0) | 26,617 (24.0) | <0.001 |
| Loop diuretic | 41,169 (6.9) | 7,717 (7.0) | <0.001 |
| Other anti-hypertensive agents^10^ | 58,503 (9.8) | 12,028 (10.9) | <0.001 |
| Anti-arrhythmic medications | 21,710 (3.7) | 3,662 (3.3) | <0.001 |
| Antithrombotic | 19,619 (3.3) | 3,399 (3.1) | <0.001 |
| Antipsychotic | 16,001 (2.7) | 3,995 (3.6) | <0.001 |
| Dopamine agonist | 3,566 (0.6) | 776 (0.7) | <0.001 |
| Peripheral vascular disease medications^11^ | 2,570 (0.4) | 382 (0.4) | <0.001 |
| Anti-smoking medications | 25,841 (4.3) | 5,714 (5.2) | <0.001 |
| Non-statin lipid lowering medications | 59,670 (10.0) | 8,785 (8.0) | <0.001 |
| **Cardiovascular procedures** | | | |
| Electrocardiography | 103,775 (17.4) | 20,690 (18.8) | <0.001 |
| Echocardiography | 30,270 (5.1) | 5,926 (5.4) | <0.001 |
| Stress test | 15,763 (2.7) | 2,704 (2.5) | <0.001 |
| Cardiac catheterization | 1,693 (0.3) | 115 (0.1) | <0.001 |
| Percutaneous coronary intervention | 1,493 (0.3) | 41 (0.04) | <0.001 |
| Coronary artery bypass graft surgery | 49 (0.01) | 1 (0.0) | 0.008 |
| Pacemaker/defibrillator implantation | 497 (0.1) | 85 (0.1) | 0.50 |
| Peripheral arterial revascularization procedures | 44 (0.01) | 8 (0.01) | 0.96 |
| **Duration of Follow-up in days** | 2430 (1092) | 1467 (1105) | <0.001 |
| Values expressed as numbers (%) unless stated otherwise  Abbreviations: ACEI: Angiotensin converting enzyme inhibitors; ARB: Angiotensin-receptor blockers; DPP-4: Dipeptidyl peptidase 4 inhibitors; eGFR: estimated glomerular filtration rate using the Modification of Diet in Renal Disease (MDRD) Study equation; GLP-1: Glucagon-like peptide 1 agonists; SGLT2 = Sodium glucose cotransporter 2 inhibitors;   1. Family history of cardiovascular disease was defined using ICD-9-CM codes (supplement) 2. Smoking as defined using ICD-9-CM codes: 3051 and V1582. 3. Diagnoses & procedures as defined by the Agency for Health Research and Quality Clinical Classifications Software disease categories (AHRQ-CCS). 4. Diagnosis using ICD-9 or CPT codes as defined in prior studies (Supplement). 5. Diagnosis is based on selected ICD-9-CM diagnosis codes from category 56 of AHRQ-CCS Supplement). 6. Malignancy, metastatic neoplasm, and Acquired Immunodeficiency Syndrome were defined using Deyo et al method in calculating the Charlson comorbidity index. 7. The Charlson comorbidity total score was calculated using Deyo et al method. 8. Cardiovascular risk was calculated using D’ Agostino et al method for calculating the Framingham risk score. 9. Approximately, only half of the cohort was diagnosed with diabetes before the index date.   10. Other anti-hypertensive agents include α-blocker medications, clonidine, α-methyldopa, hydralazine, minoxidil, and reserpine  11. Peripheral vascular disease medications include pentoxiphylline, cilostazole, papaverine, tolazoline, cyclandelate, and ethaverine  12. Results for total cholesterol were available for only 80,718 statin users and 80,821 control subjects  13. Results for HDL-cholesterol were available for only 78,111 statin users and 78,105 control subjects | | | |

| **S5 Table. Comparisons of changes in vital signs and laboratory values from baseline to follow up periods in propensity score matched cohort of statin users and nonusers** | | | |
| --- | --- | --- | --- |
|  | **Statin-users**  **N=81,146** | **Active comparators**  **N=81,146** | **p-value** |
|  | Results | Results |  |
| Difference in systolic BP (mmHg) in follow up in comparison to baseline^1^ | | | |
| Mean (SD) | -2.6 (13.0) | -1.7 (12.5) | <0.001 |
| Median (interquartile) | -2.0 (-9.8, 5.4) | -1.1 (-8.8, 6.0) | <0.001* |
| Difference in diastolic BP (mmHg) in follow up in comparison to baseline^2^ | | | |
| Mean (SD) | -1.9 (7.8) | -1.3 (7.7) | <0.001 |
| Median (interquartile) | -1.6 (-6.5, 3.0) | -1.1 (-5.8, 3.5) | <0.001* |
| Difference in mean LDL-cholesterol (mg/dL) in follow up in comparison to baseline^3^ | | | |
| Mean (SD) | -25.2 (31.5) | -0.9 (23.6) | <0.001 |
| Median (interquartile) | -24.0 (-45.4, -4.0) | -1.3 (-13.8, 11.6) | <0.001* |
| Difference in lowest recorded LDL-cholesterol (mg/dL) in follow up in comparison to baseline^4^ | | | |
| Mean (SD) | -42.5 (37.3) | -14.6 (27.7)^4^ | <0.001 |
| Median (interquartile) | -41.0 (-66.0, -16.0) | -14.0 (-30.0, 1.0) | <0.001* |
| Difference in highest recorded LDL-cholesterol (mg/dL) in follow up in comparison to baseline^5^ | | | |
| Mean (SD) | -3.9 (38.1) | 13.3 (29.0) | <0.001 |
| Median (interquartile) | -3.6 (-27.0, 17.0) | 11.0 (-2.8, 27.0) | <0.001* |
| * Comparison of median and interquartile was performed using Wilcoxon rank-sum (Mann-Whitney) test  1. This parameter was calculated as the mean and standard deviation of (difference between mean of all systolic blood pressure readings throughout the follow up period in each individual patient and her/his mean of all systolic blood pressure readings throughout the baseline period)  2. This parameter was calculated as the mean and standard deviation of (difference between mean of all diastolic blood pressure readings throughout the follow up period in each individual patient and her/his mean of all diastolic blood pressure readings throughout the baseline period)  3. This parameter was calculated as the mean and standard deviation of (difference between mean of all LDL-Cholesterol throughout the follow up period in each individual patient and her/his mean of all LDL-Cholesterol throughout the baseline period)  4. This parameter was calculated as the mean and standard deviation of (difference between lowest measured value of all LDL-Cholesterol throughout the follow up period in each individual patient and her/his lowest measured value of all LDL-Cholesterol throughout the baseline period)  5. This parameter was calculated as the mean and standard deviation of (difference between highest measured value of all LDL-Cholesterol throughout the follow up period in each individual patient and her/his highest measured value of all LDL-Cholesterol throughout the baseline period) | | | |

| **S6 Table. Summary of the major placebo controlled randomized cardiovascular outcome trials evaluating statins in a primary prevention in patients with diabetes^1^** | | | | | | |
| --- | --- | --- | --- | --- | --- | --- |
| **Trial name,**  **Year** | **Patients No** | **Median/ average FU**  **(years)** | **Target population & other Comments** | **Main results of primary outcome** | **Did total mortality decrease?** | **Drop-out/ quit taking the study drugs** |
| **ASCOT-LLA** (reference 73 in the manuscript)[_ENREF_78](#_ENREF_78) | 2,532 | 3.3 | Substudy of a larger study (10305 patients) of patients with hypertension, average or lower hypercholesterolemia, and ≥ 3 CVD risk factors. | HR of MACE = 0.77, (95%CI 0.61– 0.98) | Not reported | 13% of statin group stopped statin; 9% of control group used statins. 306 (12%) had preexisting CVD |
| **ASPEN**  (reference 74 in the manuscript) | 1905 | 2.4^2^ | Substudy of a larger study that included secondary and primary prevention | HR of MACE = 0.97 (95%CI = 0.74-1.28) | Not reported | 33% discontinued for various reasons |
| **CARDS** (reference 75 in the manuscript) | 2838 | 3.9 | All patients had diabetes with a diabetes complication, smoking, or hypertension, but without high LDL-C levels | Rate reduction of MACE = 37% (95%CI = -52 to -17); HR of total mortality = 0.68 (95%CI = 0.52-1.01) | No | 15% of statin group stopped taking the statin; 9% of placebo group were taking statins |
| **HPS** (reference 76 in the manuscript) | 2912 | 4.8 | Substudy of a larger study that included secondary and primary prevention of high-risk patients with vascular diseases or diabetes. | Rate reduction of MACE = 33% (95%CI: 17-46%). | Not reported | Approximately 15% were not taking study medication & up to 25% of placebo started staking statins |
| CVD = cardiovascular disease; FU = Follow up in years; hs-CRP = high-sensitivity C-reactive protein, HRT = hormone replacement therapy; LDL-C = LDL-Cholesterol; M = men; W = women. MACE = major acute cardiac events (the definition of MACE varied in different studies), RR = relative risk  ASCOT-LLA = Anglo-Scandinavian Cardiac Outcomes Trial--lipid-lowering arm; ASPEN = the Atorvastatin Study for Prevention of Coronary Heart Disease Endpoints in non-insulin-dependent diabetes mellitus; CARDS = the Collaborative Atorvastatin Diabetes Study; HPS = Heart Protection Study  1. We defined major randomized controlled trials as trials with at least 1000 participants, placebo-controlled, continued for at least 6- months, and had a clinical primary outcome (not a laboratory or imaging measurement). These studies were the studies listed by the American Diabetes Association (ADA) in the 2008 Standards of Medical Care in Diabetes, and identified in more recent systematic reviews (references 66,71,72 in the manuscript)  2. Median follow-up duration of the study was 4 years for the overall study but the primary prevention arm participants were recruited in the 2^nd^ and 3^rd^ years of the study. | | | | | | |
