# Supplementary material for: Statins and renal disease progression, ophthalmic manifestations, and neurological manifestations in veterans with diabetes: A retrospective cohort study
Source: PLoS One. 2022 Jul 21;17(7):e0269982. doi: 10.1371/journal.pone.0269982 (PMC9302779; doi:10.1371/journal.pone.0269982)
Supplement: Attachment — Submitted filename: JE comments.docx [file pone.0269982.s002.docx]

**Statins and renal disease progression, ophthalmic manifestations, and neurological manifestations in Veterans with diabetes: A retrospective cohort study**

**Comments**:

This is an interesting article that provides valuable information on potential adverse events of statin use in diabetic patients. Authors conclude that among patients with diabetes, statin use is associated with a modest but significant increased risk of renal, ophthalmic and neurologic manifestations.

It is possible that authors underestimate the real magnitude of the association due to a number of reasons, namely,

- Not all patients had diabetes at index date. In fact, only 52% of patients were diabetic at baseline. This may have contributed to underestimation of the statin effects due to lower exposure time. Probably it would have been more informative if all patients had been diagnosed with diabetes at study entry.

- Authors state that different bias may be present in this type of study. On one hand, statin use may be falsely associated with better outcomes because of healthy-user bias, and being a surrogate for higher quality of care, or better access to healthcare. Alternatively, statin use may be falsely associated with worse outcomes because of more exposure to healthcare resulting in ascertainment bias or confounding by indication. In order to address these methodological concerns, authors employed a new user design with active comparators (H2 blockers or PPIs).

However, PPIs are associated with increased risk of renal disease (1)(2)(3)(4)(5), neurological (6)(7)(8) or ophthalmic manifestations (9)(10). This means that if statins had been compared to a drug unrelated to these adverse events, observed differences would have probably been higher than reported.

- At study entry, some 8% of patients had already developed the primary endpoint. The baseline characteristics table shows that at inclusion in the study, some patients with diabetes had also renal disease (1%), ophthalmic (2%) or neurological manifestations (4.5%). For the evaluation of incident events, these patients should have been excluded since that had already developed the study outcome. Authors showed results comparing both groups in a “*Healthy Cohort*” as a sensitivity analysis. A higher association between statin use and renal, ophthalmic and neurological manifestations was observed.

Interestingly, there is a dose-dependent association being the incidence of adverse events much higher if intensive cholesterol lowering compared to low-moderate lowering. The article provides no information about exposure time to statins. It would have been very informative to stratify results according to statin duration.

This article offers valuable information that suggests further research on renal, ophthalmic and neurologic manifestations of statin use is warranted.

References:

1.	Al-Aly Z, Maddukuri G, Xie Y. Proton Pump Inhibitors and the Kidney: Implications of Current Evidence for Clinical Practice and When and How to Deprescribe. Am J Kidney Dis [Internet]. 2020;75(4):497–507. Available from: https://doi.org/10.1053/j.ajkd.2019.07.012

2.	Fontecha-Barriuso M, Martín-Sanchez D, Martinez-Moreno JM, Cardenas-Villacres D, Carrasco S, Sanchez-Niño MD, et al. Molecular pathways driving omeprazole nephrotoxicity. Redox Biol [Internet]. 2020;32(December 2019):101464. Available from: https://doi.org/10.1016/j.redox.2020.101464

3.	Guedes JVM, Aquino JA, Castro TLB, De Morais FA, Baldoni AO, Belo VS, et al. Omeprazole use and risk of chronic kidney disease evolution. PLoS One. 2020;15(3):1–16.

4.	Ness-Jensen E, Fossmark R. Adverse Effects of Proton Pump Inhibitors in Chronic Kidney Disease. JAMA Intern Med. 2016;176(6):868.

5.	Wu B, Li D, Xu T, Luo M, He Z, Li Y. Proton pump inhibitors associated acute kidney injury and chronic kidney disease: data mining of US FDA adverse event reporting system. Sci Rep [Internet]. 2021;11(1):1–8. Available from: https://doi.org/10.1038/s41598-021-83099-y

6.	Makunts T, Abagyan R. How can proton pump inhibitors damage central and peripheral nervous systems? Neural Regen Res. 2020;15(11):2041–2.

7.	Makunts T, Alpatty S, Lee KC, Atayee RS, Abagyan R. Proton-pump inhibitor use is associated with a broad spectrum of neurological adverse events including impaired hearing, vision, and memory. Sci Rep [Internet]. 2019;9(1):1–10. Available from: http://dx.doi.org/10.1038/s41598-019-53622-3

8.	Novotny M, Klimova B, Valis M. PPI long term use: Risk of neurological adverse events? Front Neurol. 2019;10(JAN).

9.	Hanneken AM, Babai N, Thoreson WB. Oral proton pump inhibitors disrupt horizontal cell-cone feedback and enhance visual hallucinations in macular degeneration patients. Investig Ophthalmol Vis Sci. 2013;54(2):1485–9.

10.	Schönhöfer PS. Wernera B, and Tröger U. Ocular damage associated with proton pump inhibitors. BMJ 1997;314:1805
